# Hidden Markov Model-Based Smart Annotation for Benchmark Cyclic Activity Recognition Database Using Wearables †

**DOI:** 10.3390/s19081820

**Published:** 2019-04-16

**Authors:** Christine F. Martindale, Sebastijan Sprager, Bjoern M. Eskofier

**Affiliations:** 1 Machine Learning and Data Analytics Lab, Computer Science Department, 91052 Erlangen, Germany; bjoern.eskofier@fau.de; 2 Faculty of Computer and Information Science, University of Ljubljana, 1000 Ljubljana, Slovenia; sebastijan.sprager@fri.uni-lj.si

**Keywords:** activity recognition, benchmark database, gait analysis, inertial measurement unit, gait phases, cyclic activities, home monitoring, smart annotation, semi-supervised learning

## Abstract

Activity monitoring using wearables is becoming ubiquitous, although accurate cycle level analysis, such as step-counting and gait analysis, are limited by a lack of realistic and labeled datasets. The effort required to obtain and annotate such datasets is massive, therefore we propose a smart annotation pipeline which reduces the number of events needing manual adjustment to 14%. For scenarios dominated by walking, this annotation effort is as low as 8%. The pipeline consists of three smart annotation approaches, namely edge detection of the pressure data, local cyclicity estimation, and iteratively trained hierarchical hidden Markov models. Using this pipeline, we have collected and labeled a dataset with over 150,000 labeled cycles, each with 2 phases, from 80 subjects, which we have made publicly available. The dataset consists of 12 different task-driven activities, 10 of which are cyclic. These activities include not only straight and steady-state motions, but also transitions, different ranges of bouts, and changing directions. Each participant wore 5 synchronized inertial measurement units (IMUs) on the wrists, shoes, and in a pocket, as well as pressure insoles and video. We believe that this dataset and smart annotation pipeline are a good basis for creating a benchmark dataset for validation of other semi- and unsupervised algorithms.

## 1. Introduction

Activity monitoring using wearables is ubiquitous in daily life and is beginning to be used for medical purposes, from level of sedentary behavior to gait analysis [1]. For home monitoring, rehabilitation, and health outcome measurements, accurate step counts and cycle analysis within realistic environments is needed. Commercially available activity monitors are currently used for some health outcome studies, often using daily step counts as a measure [2]. However, the accuracy of these devices depends on location and speed [1,3,4]. Furthermore, the algorithms used are often proprietary, with unknown accuracy, and subject to regular updates with an unspecified change in performance. For research and medical purposes, algorithms with known accuracy are needed.

To understand the limitations of algorithms and compare them fairly, one needs benchmark datasets. There are several public databases within the activity recognition field which use wearables; however they focus on the overall activities, often on a very high level [5], whereas for applications such as home monitoring and rehabilitation cycle level information is needed. Furthermore, they often focus on steady-state activities [6] and are performed under laboratory conditions [7]. For gait analysis, the databases are often also only collected under laboratory conditions, only steady state, with few subjects [8,9] or with low repetitions per activity [10]. Finally, within the home monitoring field, the data is often without labels or only sporadic and self-annotated ones [11].

One of the barriers to providing benchmark datasets with cycle level information is the effort required to obtain and annotate them. Ontologies for daily activities, such as cooking [12,13], have been used simplify to the task when looking at non-cyclic data. Semi-supervised learning is also a common approach to reduce the labeling effort for activity level labels [14,15]. We propose a method which will reduce the annotation effort where cycle phase labels are needed. Cycle phases is a more general term for gait phases, i.e., swing and stance phase, when referring to cyclic motions other than walking which involve alternating ground contact and in the air phases of the foot.

Ideally a benchmark dataset would be collected under free living conditions; however, the ability to provide accurate, cycle level labels under such conditions is still an open problem. Therefore, a compromise between free living and laboratory environments needs to be found. This can be achieved by having task-driven protocols where the precise start, stop and manner of performing the activity is left to the participant’s interpretation of an instruction. However, using a protocol and environment where collecting and labeling ground truth data is still feasible.

We provide a dataset, and data collection protocol, which addresses these needs. This dataset is an extension of the smaller one published in [16], using a similar protocol. In contrast to existing datasets, ours includes transitions between activities, initiation, and termination of them, varying lengths of bouts and examples of weight change which are not considered to be walking. The sensor modalities used are inertial measurement units (IMUs) at several body locations, pressure insoles, and videos. To provide cycle phase labels for this dataset, we propose a smart annotation pipeline based on the edge-detection method from [16], which is extended in this paper using an iteratively trained hierarchical hidden Markov model (hHMM). We also compared local cyclicity estimation [17] and peak detection for the labeling of one of the activities where the other methods were insufficient. The data is labeled on three levels; protocol, basic activity, and cycle phase level. We analyze the cost of these smart annotation methods relative to that of complete manual labeling and investigate for which activities they are most effective. The aim of this protocol, pipeline and dataset is to create a coherent and publicly available large-scale benchmark database for the training and testing of algorithms for home and health monitoring by using and testing smart annotation methods.

We provide a novel public dataset which is diverse in sensors and activities, which focuses on cycle phases and transitions as well as activities. We also develop a novel smart annotation pipeline, reusable protocol, and definitions. The dataset and pipeline are extensions of [16], a workshop paper by the authors. In [16], the edge-detection method was proposed and a dataset of 20 subjects was published. This method relied on pressure data. In this paper, the pressure data was unusable with this method, so to extend the dataset a new method was proposed using IMU data, namely the iterative training of the hHMM and the use of the local cyclicity estimation for cycle detection where no phases occurred. The dataset itself was extended from 20 subjects to 80 and is available at activitynet.org.

## 2. Related Work

### 2.1. Smart Annotation Approaches

Methods used to reduce the cost of labeling data can be grouped into several categories: shared, transfer, and unsupervised learning as well as active, population, and semi-supervised labeling. The three learning methods refer to the way models are trained to provide labels. The three labeling methods refer to when only partial data within a dataset is labeled and how this is then used to label the remaining data.

Shared learning combines datasets where not all datasets contain all activities [18]. Transfer learning uses labeled data in the source domain to train a model for use in the target domain, hence models are shared across domains [19]. In contrast unsupervised learning attempts to label data without using prior knowledge, by identifying the underlying structure of the data. To apply unsupervised approaches, one needs to define to which labels these underlying structures refer and the applicability of them within the particular application domain. Unsupervised approaches successfully used in the wearables domain include clustering methods [20], cross-correlation-based ones [17] and information gain-based methods [21]. Part of our data was completely unlabeled and so we used a basic peak detector and compared it to the local cyclicity estimator, the cross-correlation-based unsupervised method from [17].

Active labeling refers to automatically extracting and manually labeling sections of the data which would be most effective to annotated or where labeling errors are most likely to have taken place. Liu et al. identified the most informative samples by identifying those sections where the classifier outputs conflicted or where they had the lowest confidence [22]. Hossain et al. combine an unsupervised clustering approach with active learning for the labeling of smart home data which allowed new activities and clusters to be dynamically added [23]. On the other hand, population labeling is the annotation of subsets of data, where these are chosen to represent certain groupings of the population. This could be by age, gender, disease stage, or type. For example, [24] uses gender and physical characteristics to find a matching model, and in [25] a new user is matched to the most similar model from the existing trained models. Semi-supervised annotation is where a labeled subsection of a dataset is used as training data for a model which suggests labels for the remainder of the data. Diete et al. used IMU data synchronized to video data to partially label a multi-modal dataset [26]. This data was then used to create templates and dynamic time warping was applied to give the labeler suggestions for the remaining data [14,26]. Active learning and semi-supervised annotation are combined by Alemdar et al., who use the actively labeled data from a smart home to iteratively train an HMM for activity recognition [27].

In this paper, we used the output of an edge-detection method for semi-supervised annotation, developed in previous work [16]. The resulting data we use to iteratively train a hierarchical HMM (hHMM). The main difference is that we are interested in both activity and cycle information where both classification and segmentation are necessary, hence the need for a hierarchical model.

### 2.2. Cyclic Activity Datasets

Activity recognition is a well-established field with a variety of public databases. However, most of these datasets focus on video and environmental sensor data and focus on labeling complex activities such as house work or cooking. For applications such as home monitoring or rehabilitation, more basic activities are useful, such as walking. Furthermore, these basic activities should be accurately characterized in terms of duration, quality, and variability. In this paper, we focus on basic cyclic activities such as walking, running, and jumping. Although in the case of home monitoring of patients with motion disorders, we would not expect jumping data, we may do so in the case of sports rehabilitation. Including a variety of cyclic activities would also give insight into how generalizable algorithms are to unknown, but cyclic data such as abnormal, but nonetheless cyclic motion (e.g., walking with a limp). Specifically, we are focusing on data where exact cycle parameters could be extracted. Such data would allow the comparison and analysis of algorithms proposed for home monitoring and rehabilitation purposes.

Public datasets containing wearable sensor data and labels for cyclic activities often isolate the steady-state sections of behavior, such as [6,28]. This excludes transition stage data which is unavoidable in realistic scenarios. Furthermore, traditional activity recognition databases usually only provide labels for the overall activity, rather than individual cycles [5,28]. The databases where cycle information is given can be split into two categories: those with step counts or labeled cycle borders [29,30,31,32,33] and those with labeled heel-strike and toe-off events [8,9]. These databases focus on steady-state gait and have under 20 subjects each. One database focusing on gait, with more than 700 participants [10], is unfortunately limited to less than 20 strides per subject and cycle borders or gait events are not published with the database, only the activity, namely gait, slope, and stair. Furthermore, the data is only from waist mounted sensors which limits the application domain. In all the cases where gait events are given, the data is taken from a limited capture volume due to using a ground truth such as motion capture [8] or using treadmills [9]. While [30] had 175 subjects, the data was of a 20 m straight walk repeated 6 times and they used only accelerometer data from one side of the body. Although [29] had outdoor motion data with varied speeds and sensor locations, they also only focused on walking. While [31,32] had outdoor walking and running or jogging instances, they both had data from under 20 participants. The Digital Biobank [33] offers data from 70 patients; however only the straight strides are labeled. One wearables database where a variety of cycles are present, not just running and walking, is [7]; however, the activities are limited to the capture volume of a motion capture system and the cycle counts are not given. In conclusion, the available datasets are often unrealistic or lack subject numbers or repetitions. The databases mentioned here are described in more detail in Table 1 and Table 2, where the final entry is a description of the dataset presented in this paper, which will be detailed in the following section. There exist more databases with wearable sensor data; however, to the best of the authors’ knowledge there are none with cycle level labels under such realistic conditions.

## 3. Dataset

### 3.1. Goals of the Dataset

To address the gaps in the publicly available datasets, we provide a protocol and dataset to fulfil these criteria:

- (Pseudo) realistic
- Cyclic activities
- Cycle and activity level annotations
- Initiation and termination of activities
- Variety of bout durations
- Task orientated
- Wearables sensors at common locations
- Non-straight walking
- Transition phases between activities

By fulfilling these criteria the dataset could then be used to investigate generic cyclic activity algorithms, validate semi-supervised and home monitoring methods, as well as understand their limitations and normal behavior during edge cases, such as unclear start of bouts and failed or aborted tasks. The main challenges for such a dataset collection are practical concerns such as the need for many subjects, repetitions, and creating tasks as realistically as possible, while still being able to create cycle level annotations for them. The annotation challenges include the sheer volume of data to be labeled and definitions, such as the start of activities or the labeling of unclear ones. This is normally avoided by providing only steady-state cases or by excluding unclear activities; however, in a real-world environment these cases can be expected. To the best of the authors’ knowledge, there are no existing wearables databases which have phase level labels in pseudo realistic settings with a variety of cyclic activities, as well as a variety of sensor locations.

### 3.2. Dataset Description

We collected data from 80 subjects who all gave written, informed consent. This study was approved by the ethics committee of the Friedrich-Alexander-Universität Erlangen-Nürnberg, No. 106_13B. The physical characteristics of the subjects are shown in Table 3. The data collection was carried out at the Friedrich-Alexander University Erlangen-Nürnberg, Erlangen, Germany and at the Faculty of Computer and Information Science, University of Ljubljana, Ljubljana, Slovenia; Respectively 56 and 24 subjects were collected at each location. Data from 20 of the subjects collected in Slovenia was previously published in [16]. The dataset is available at www.activitynet.org.

**Figure 1:**
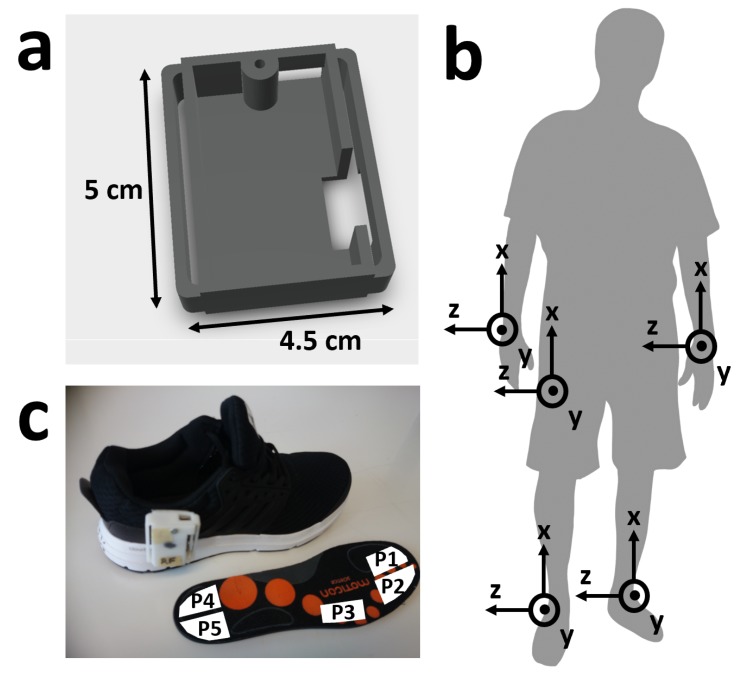
Sensor type and location. (**a**) Photograph of IMU sensor system in 3D printed case. (**b**) Diagram showing sensor attachment to shoe, using industrial Velcro and Moticon insole, which was used instead of the original sports shoe insole pressure sensor location and approximate size and shape within the insole. Also showing axes location for the insole accelerometer. (**c**) IMU sensor locations on the body with corresponding axes.

Each subject wore 5 IMU sensors; one on each wrist, one on the lateral side of each shoe and, where possible, one in a trouser pocket, preferably the right front pocket. Due to technical failures, 6 subjects do not have data from the pocket IMU sensor, and 1 subject does not have from one wrist sensor. The IMUs were part of a Bosch development platform. Each IMU measured acceleration (±8 g) and angular velocity (±2000 dps) with a frequency of 200 Hz. The sensors were calibrated at the end of each day, according to the method given in [34], where the accelerometer and gyroscope were calibrated using six static positions and a complete rotation about each of the three axes. The placement, attachment mechanisms and orientation of the sensors are shown in Figure 1. All sensors were synchronized by a simultaneous, single packet Bluetooth-based clock reset and start command. Due to technical failure, the IMU synchronization failed for 2 subjects.

Each subject also wore Moticon pressurized insoles. Moticon Science Software version 01.10.00 was used for sensor connection, download of the data and synchronization with the camera system [35]. Due to limited Moticon insole sizes, subjects were restricted to shoe sizes of between 38 and 44. Each insole recorded 5 pressure sensors and a 3-axis accelerometer at 100 Hz. For the 56 subjects collected in Germany, there was significant drift in the pressure signals and random data package loss. However, the automatic zeroing feature was available at 100 Hz for the 24 subjects collected in Slovenia which corrected both problems. The Moticon data was synchronized to the cameras using a time stamp-embedded QR code displayed within the Moticon software. The calibrated IMUs were then synchronized to the Moticon data by performing a piecewise cross correlation between the x-axis acceleration of the insole and the shoe-mounted IMUs. We assume that the acceleration of the insole is the same as that of IMU mounted to the same shoe. The Moticon acceleration data was re-sampled with a range of ratios between 1.4 and 3.3 with a resolution of 0.001. The ratio with the highest cross correlation value was then chosen as the re-sampling factor, usually close to 2.0. The time difference achieving the highest cross correlation was then selected and used as the lag between the insoles and the IMUs. This was performed separately for each section, subject, and foot. Due to technical failure, the Moticon to IMU system synchronization failed for 1 subject.

As reference for the activities, the data was recorded using static cameras with a view of each location and one camera held by the researcher which was focused on the subjects’ feet. The combination of hand held camera and room camera minimized occasions of occlusion.

### 3.3. Data Collection Protocol

**Figure 2:**
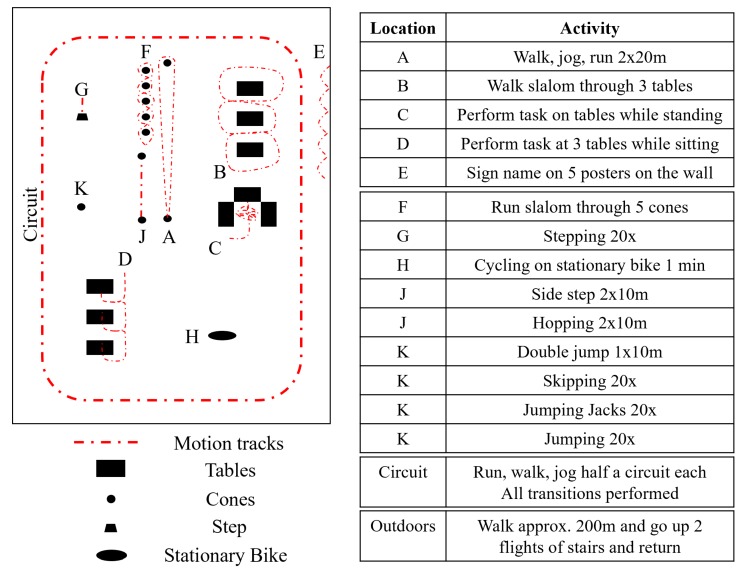
Layout of the activities (Germany). The starting point of each activity is shown by the letter whose key is in the table on the right. Double lines in the table separate the 4 sections of activities.

The overall structure of the data collection was always the same; however, the individual activities within each section were given in a randomized order per participant. Each activity had an assigned location with a marked starting point as shown in Figure 2. There were 4 sections of activities. Between each section the sensors and cameras were stopped, restarted, and resynchronized. The participant was then instructed to jump 3 times and lift each foot into the air. At the end of each section this procedure was repeated. This was used as a manual synchronization method, to check for errors in the other synchronization methods. During data collection in Erlangen, only the cameras were restarted between each section; however the synchronization jumps were still present. The complete recording took about 30 min with another 15 min for setup and instruction.

The first section of activities included: walking, jogging, and running, each performed as 2 times 20 m bouts, walking a slalom route through 3 tables spaced 2 m apart, sorting cards on 3 tabletops while standing, sitting down and performing three tasks at three different tables (sorting cards, filling out a form and relaxing) and signing one’s name on 5 posters spaced 2 m apart. Each activity was performed at a fixed location; however, as the tasks were in a random order, the length of walking and standing bouts between activities varied. Jogging was defined as “as one would jog for exercise in the evening” and running as “as if one is late for a bus”.

The second section involved motion around a 40 m by 20 m circuit. Running, jogging, and walking were alternated such that each transition between the three activities occurred once. The transitions occurred after each half a circuit. The range of permutations of these transitions were also randomized per participant.

The third section involved a variety of cyclic activities; running slalom through 5 evenly spaced cones (2 times 10 m), stepping 20 times onto and off a step, cycling for one minute on a stationary bicycle, side-stepping for 2 times 10 m (alternating front foot), hopping 2 times 10 m (alternating hopping leg), jumping with both feet together over a line for 10 m, skipping 20 times, performing jumping jacks 20 times and jumping on the spot 20 times. At the location in Slovenia a stationary bicycle was unavailable, therefore the activity was replaced; participants ran on the spot with high kicks 20 times. Activities such as skipping were not always successful; however, the participant would try up to three times. If an activity was performed incorrectly (e.g., stepping over the step instead of onto it), then it was simply restarted. During the final section, the participant walked to the neighboring building, up and down two flights of stairs and then returned.

Figure 2 refers to the layout for the data collected in Germany. For that from Slovenia, the first section was performed in a classroom and the adjacent corridor; however, the setup for each individual task was replicated. The circuit was performed outdoors and so the stairs and outdoor walking section was performed between the classroom and an outdoor circuit location.

The smart annotation tool introduced by [16] was extended for the labeling of this dataset. The study protocol was imported per subject and the exact timing of the start of each activity adjusted within the tool using the synchronized video and pressure data. The labeling of cycles and cyclic phases was performed using the various smart annotation approaches and label definitions which are detailed in Section 4. The results of these smart annotation approaches were then manually corrected using the visualization of left and right normalized shoe-mounted IMU data and insole sensor data synchronized to each other and to the hand held camera video stream. For both cycle and activity labeling one could add or remove a segment, and change the label of a segment.

## 4. Annotation

### 4.1. Activity and Label Definitions

Another problem with realistic datasets is the choice of definition for activity bouts or of when a change of weight is a step and when not. These problems are circumvented in many public databases by only labeling steady-state regions; however, they occur in daily life and are abundant in this dataset. In this section, we will describe our labeling conventions.

Each of the tasks mentioned in Figure 2 could contain several basic activities as specified in Table 4. Rest refers to all data that is not covered by any other label, and includes standing and weight shift, except for periods of video occlusion or where one or more sensor fell off which were labeled as unknown. This definition enables the calculation of statistics about all non-rest activities where we are sure the labels are clear. Sit and rest are simple activities and contain no phases or repetitions. All the other basic activities are cyclic and contain two phases; on the ground and off the ground. Here we will refer to these as stance and swing phases, respectively. These cycle phases are defined per foot. One exception is cycling where the pressure data was often insufficient to define cycle phases and therefore only cycles are defined.

All activities transitioning to or from rest (aka standing), begin with a swing phase. This defines that an activity starts when the foot leaves the ground for the first time in a sequence and ends when it touches the ground the final time in that sequence. This means that at the start of a bout, one foot will still be in rest when the other starts the activity. Sequences of activities will be referred to as bouts. If there is a transition between two non-rest activities, then there could be a stance phase separating them. This definition was used so that even sensor data from just one sensor could be isolated with the correct labels.

At the start or end of a bout there are sometimes a few abnormal movements, e.g., the first step when standing up from chair or when changing directions before starting to walk. These movements were excluded if, by video or signal inspection, these cycles could not be said to be part of the bout or in the same direction as the bout (e.g., the angle of the foot changes in mid swing when standing up from a chair). The reason for this style of labeling is the need to calculate bout information from the ground truth labels. This is a less clear definition but a necessary one to exclude weight shifts from bouts, as well as cases where they are simply isolated movements but could not be said to be a part of the bout. It can also often be simplified by saying that only the initial and terminal stride may begin with the feet together.

All methods used data from the sensors located at the feet because on-the-ground and off-the-ground phases needed to be labeled. The labels for the remaining sensors were then inferred due to the synchronization of all systems.

### 4.2. Smart Annotation Methods

The final dataset consisted of about 30 h of data, where one phase was on average 0.5 s long. The only feasible way to label such a dataset was by smart annotation methods. The data from the first 20 subjects was labeled using the change in pressure data due to initial and final ground contact of each foot. This change in pressure was found by an edge-detection method. The pressure data from the remaining subjects had drift and occasional data loss problems, therefore a smart annotation method, independent of pressure data was needed. We used the data from the 20 labeled subjects to train a hierarchical hidden Markov model (hHMM). This model we used to predict the next 10 subjects. These predictions were manually corrected, and the resulting labels used to update the hHMM, which in turn was used to predict the next 10 subjects’ data. This was repeated until the complete dataset was labeled. The only exception was data for cycling where the pressure data was insufficient to be used as a ground truth and so only the cycles were annotated. This was done by comparing an unsupervised method to a general peak detection method. Between applying these smart annotation methods and manually correcting the data a few post-processing steps were applied to conform to the definitions from Section 4.1. Manual labeling was performed with an updated version of the smart annotation tool described in [16].

#### 4.2.1. Edge Detection

In previous work we compared two edge-detection methods, a proprietary Moticon algorithm and a basic thresholding method [16]. The edge-detection method based on the rising and falling edges of each individual pressure sensor was found to be superior. (This method was referred to as EdgeDet1 in [16].)

The positive and negative peaks of the derivative of the filtered, normalized pressure data were found. These peaks were then filtered using a set of empirically found rules. The located edges were then manually corrected by inspection of all available sensors and video streams. 17.2% of the edges processed in this manner required manual correction. The protocol labels were used to identify the activity label, whereas rest or sitting was found by an empirically found energy threshold. Further implementation details can be found in [16]. The data used for this method was the data collected in Slovenia, thus consisted of 24 subjects’ data.

#### 4.2.2. Iterative hHMM

The manually corrected labels from the first 24 subjects, processed using the edge-detection method, were used to provide training data for a hierarchical hidden Markov model (hHMM). The gyroscope sagittal plane (GZ) and accelerometer axial plane (AX) data from both shoe-mounted sensors were used for training the model. The model treated the data from each foot independently. The choice of these axes and sensors, as well as the basic HMM architecture was chosen by the success of the models used in [31,36]. Features were calculated from the raw data using a windowing approach with a window length of 70 s and step size of 5 ms. The variance, first three coefficients of the second order polynomial fit, as well as the raw data itself were used as features. All features were normalized per person to minimize inter-person differences. The features were described using a Gaussian Mixture Model (GMM) where the number of centers was trivially initialized to 4 centers per phase or simple activity. Simple activity refers to data labeled as rest or sit. The densities were calculated using ten estimation maximization (EM) iterations and a diagonal covariance matrix was used.

The transition probabilities were initialized as a left-right continuous model within each phase or activity and as a fully connected model between phases and activities. Unlike the architecture used in [31], phases are included as an additional hierarchy rather than just cycles per activity. The non-zero transitions were initialized uniformly. The simple activities were assigned 3 internal states representing initiation, steady-state, and termination phase. The complex activities were made up of one HMM per phase, each consisting of 4 internal states. This is based on the assumption that gait can be described by up to 8 phases [37]. The training of the model was semi-supervised in that the initiation of activities and phases was supervised, but the internal states were unsupervised. The unsupervised states were always initialized linearly. The hHMM was implemented using the using Java Speech Toolkit (JSTK) due to its ability to handle semi-supervised training [38].

The manually corrected output of the edge-detection method was used for training with 100 iterations before the model was used to predict the next 10 subjects. To reduce manual labeling effort, the predictions were restricted to the activities known to occur within each task, detailed in Table 4. When new data was added to the training set, the new model was initialized from the final model of the previous iteration and all labeled data from all previous iterations were used for training.

#### 4.2.3. Cycle Detection

The data from the cycling activity was unable to be initially labeled using the edge-detection method due to only minimal, or no, changes in pressure on the insole during this activity and unclear phases from the motion data. Therefore, another method to estimate the initial labels in an unsupervised manner was needed, and so a peak-detection (PD) method was compared to a local cyclicity estimation (LCE) method for cycle segmentation. The data from the cycling activity was labeled using the PD method and manually corrected. The protocol labels were used to identify the activity label, whereas rest or sitting was found by an empirically found energy threshold.

##### Peak Detection

The PD implementation used was the *findpeaks* method from MATLAB. Two thresholds were required and were found empirically using cycle data from the first subject. The PD was performed on the normalized GZ axis data where the peak at mid swing was detected. The minimum peak distance was set to 400 ms and the minimum peak height was set to 0.5 (relative to the normalized GZ data).

##### Local Cyclicity Estimator

The method and implementation of the local cyclicity estimator were used from [17]. The noise threshold was set to 1.0, the frequencies were varied from 0.5 Hz to 6 Hz. The maximum of the normalized GZ axis was detected.

#### 4.2.4. Label Post-Processing

Once a prediction by a smart annotation method was made, the following rules were automatically applied before manual correction occurred:

- Consecutive sections with the same activity and phase label were joined.
- Each activity bout could start and end only with a swing phase.

These were based on the definitions described in Section 4.1. Following manual correction, statistics such as mean and variance of cycle time and swing duration were used to highlight any outliers which were subsequently reinspected, and corrected where necessary.

#### 4.2.5. Evaluation Metrics

To evaluate the success of the smart annotation pipeline and its components we calculate the number of labels which needed to be added or deleted during the manual correction phase, with a tolerance of 50 ms, over the total number of labels according to the manually corrected reference. A label in this case referring to the segmentation point between two activities of phases. We refer to this as the effort required for labeling. We also calculate some standard metrics such as the F1 score, the miss rate and the false discovery rate which are specified below:

(1) $$F1\text{-score}=2\times \frac{\mathrm{Precision}\times \mathrm{Recall}}{\mathrm{Precision}+\mathrm{Recall}}=\frac{2\times \mathrm{TruePositive}}{2\times \mathrm{TruePositive}+\mathrm{FalsePositive}+\mathrm{FalseNegative}}$$

(2) $$\mathrm{MissRate}\;\left(\mathrm{MR}\right)=\frac{\mathrm{FalseNegative}}{\mathrm{TruePositive}+\mathrm{FalseNegative}}$$

(3) $$\mathrm{FalseDiscoveryRate}\;\left(\mathrm{FDR}\right)=\frac{\mathrm{FalsePositive}}{\mathrm{TruePositive}+\mathrm{FalsePositive}}$$

(4) $$\mathrm{Effort}=\frac{\mathrm{DeletedLabels}+\mathrm{AddedLabels}}{\mathrm{TotalManuallyCorrectedLabels}}=\frac{\mathrm{FalsePositive}+\mathrm{FalseNegative}}{\mathrm{TruePositive}+\mathrm{FalseNegative}}$$

where TruePositive refers to the case where the predicted label and the manually corrected one were within 50 ms of each other, FalsePositive refers to the case where there is a predicted label but no corresponding manually corrected one within the 50 ms tolerance window and FalseNegative refers to the case where there is no predicted label within 50 ms of a manually corrected label. TotalManuallyCorrectedLabels can also be referred to as the number of ground truth labels. The miss rate can also be referred to as the false negative rate.

## 5. Results and Discussion

### 5.1. Dataset Evaluation

**Figure 3:**
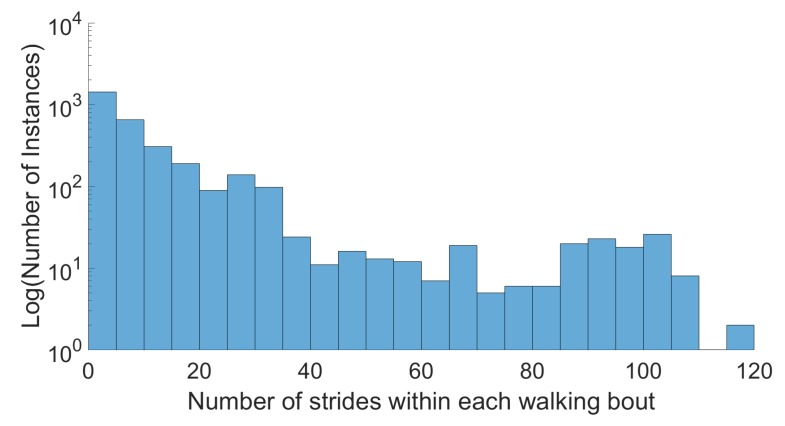
Logarithmic histogram of walking bout lengths, for all subjects.

The final dataset consisted of data from 80 subjects totaling almost 30 hours of pseudo realistic data in both indoor and outdoor settings which included 12 activities recorded using 5 IMU sensors, a pair of pressurized insoles, as well as video. All sensor data and labels can be found at www.activitynet.org, for privacy reasons video data is excluded. This data included initiation and termination sections, transitions, non-straight walking, and all inter-activity data such as weight shift. A wide range of walking bouts was achieved, as illustrated in Figure 3, as well as over 150,000 cycles, see Table 5. While there were 12 classes, the dataset was dominated by walking and rest, followed by jogging and running, as seen in Table 5.

**Figure 4:**
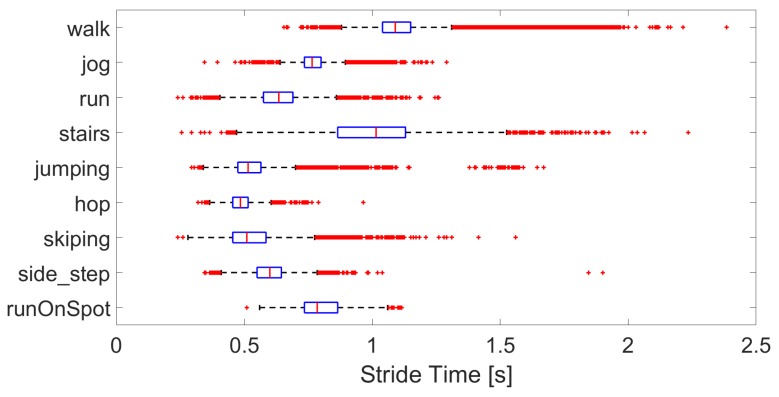
Boxplot of the stride times per activity, over all subjects.

**Figure 5:**
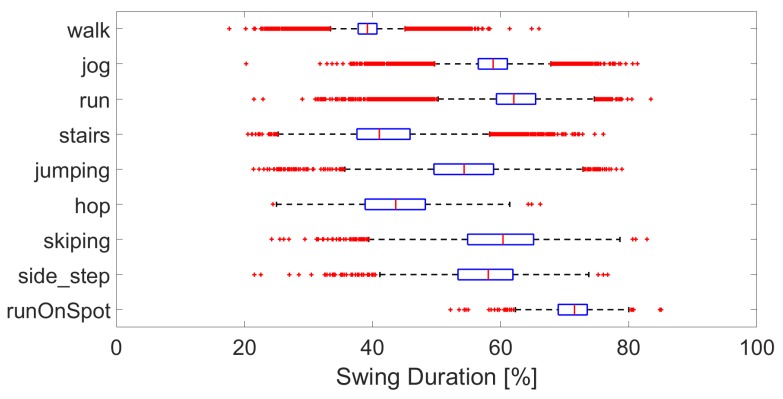
Boxplot of the swing duration per activity, over all subjects.

The characteristics of the activities are as expected with stride time decreasing between walking, jogging, and running, see Figure 4, even though the pace was self-selected. Stairs have a similar stride time to that of walking, while that of jumping, hopping, and skipping are lower. Swing duration per activity are shown in Figure 5. Again, the swing duration increases between walking and running. Skipping, side-step and jumping have a swing duration of around 60 %, while hopping, stairs and walking are closer to 40 %. While the general trends are consistent, there are many outliers, showing the wide variety of interpretations and modes of performing the various activities.

### 5.2. Smart Annotation Evaluation

**Figure 6:**
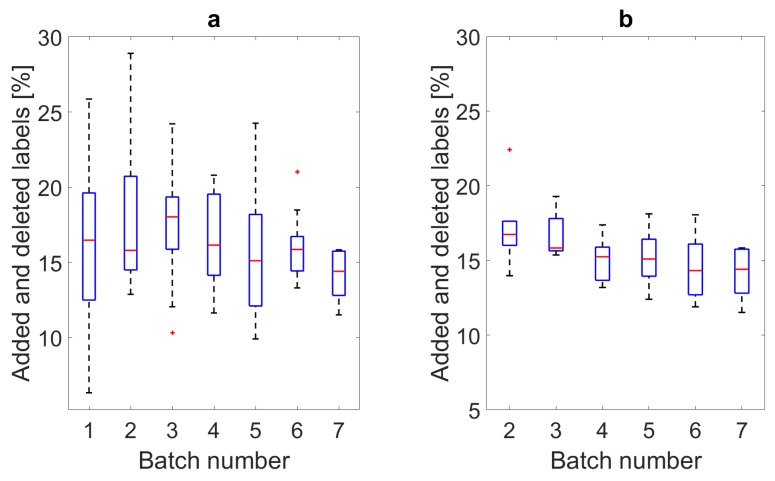
Boxplots of the effort required for manual correction of each batch of subjects using the smart annotation approaches. (**a**) Shows the actual effort per batch where Batch 1 was using the edge-detection method and Batches 2 to 7 were using the hHMM method, where the data from the previous batches is always added to the training set of the subsequent model. (**b**) Shows the labeling effort for each batch’s hHMM model when predicting the subject data from Batch 7.

The effort required for labeling each batch is illustrated in Figure 6a, the hypothetical effort for each model if the data for prediction were identical, in this case the final batch, is shown in Figure 6b. Finally, the combined effort for all smart annotation approaches combined is given in Table 6, along with the effort per activity and for the individual parts of the smart annotation pipeline. The F1 scores, miss rates and false detection rates are given in Table 7 and Table 8, respectively.

#### 5.2.1. Edge Detection

The results of the edge-detection method of [16], were post-processed according to the label definitions given in Section 4.1. Due to this, the resulting effort required for labeling decreased marginally to 16.3%. The edge-detection method was applied to the 24 subjects collected in Slovenia and is referred to as Batch 1 in Figure 6a. One can see here that there is a wide range of effort required; however, all are under 30%.

The effort required, F1 scores, miss rates and false detection rates for individual activities are summarized in the middle columns of Table 6, Table 7 and Table 8, respectively. The edge-detection method performed reasonably, at under 20% effort for most activities. It performed worst for short bout activities such as the signing posters task, walking between tables for the sitting activity and hopping. The false detection rate was generally lower than the miss rate at under 17% except for walking between activities. The miss rate was high for short bouts, hopping, and stairs. The overall F1 score was 83.8%. It was also the worst for short bouts and the best for running and jumping.

#### 5.2.2. Iterative hHMM

The manually corrected data from the edge-detection method, Batch 1, were used to train a hHMM which was used to predict the labels of the next 10 subjects, referred to as Batch 2 in Figure 6a. This data was then manually corrected, and the model trained with the updated training set, and so on until all 80 were labeled. The labeling effort required for each batch can be seen in Figure 6a. One can observe overall lowering of the effort required with each batch and a decrease in variation. In Figure 6a, the subjects per batch were different. The variation of the results reflects the variety of motion styles between subjects. To reflect the effort required by each model without the influence of different subjects per batch, each batch’s model was also used to predict the data from the final batch’s set of subjects as these were not used for the training of any of the models. These results are shown in Figure 6b.

Again, there is a general lowering of the effort and so improving of the models. The minimal decrease in effort per batch could be attributed to the effort of correcting errors in the labeling of the precise start and stop of the tasks dominating the results or a non-optimal model for the some of the activities. Alternatively, the training data is still too small in comparison to the inter-subject variation to reflect a large improvement with the given training data. One possible limitation is that some stance phases are similar to rest and the hHMM has a limited time dependency, so does not necessarily account for a long sequence of activities.

A more fine-grained illustration of the results of the hHMM-based smart annotation method per activity are given in the final column of Table 6, Table 7 and Table 8. Overall and for most walking, jogging, and running activities the hHMM method reduced the labeling effort and F1-scores considerably compared to the edge-detection method. For long bouts of steady-state walking, jogging, and running the effort is under 1% and an F1-score of over 99%. However, for some cyclic activities the effort was lower with the edge-detection method. For side-step, stairs and hopping this can be attributed to the motion consisting of two different types of motion; upstairs and downstairs and which leg is the leading leg. This could be improved by more fine-grained activity models. Furthermore, there were a variety of ways of performing a task, for example some subjects continuously jumped while others stopped between each individual jump, some subjects skipped similarly to running while others performed more of a jumping motion.

A small study was also conducted to understand the time required for labeling. The relabeling of 4 subjects was timed, where the labeler was familiar with the tools and signals. The labeling of each subject took on average 39 min ± 8 min, where the average effort was 19.8% ± 3.9%. These numbers are dependent on the familiarity of the labeler with the signals, the speed of the computer for video and GUI rendering and the number of times new motions were found and thus a more detailed inspection required.

#### 5.2.3. Cycle Detection

The results of both the PD and LCE methods were compared to the manually corrected reference. The PD method required 2.9% ± 3.9% added or deleted labels while the LCE method required an almost identical level of effort at 2.9% ± 2.8%. In both cases the cycle numbers were overestimated. The disadvantage of both methods is the need to empirically set parameters as well as a need to separately detect activity.

### 5.3. Discussion of Pipeline

While this dataset has a wide variety of cyclic activities performed at self-selected paces, it only represents participants of up to 43 years old. However, there are 80 subjects and a wide range of different interpretations of each activity. We believe that this wide variety in activity style and bout length will present challenges for the existing activity recognition and cycle detection algorithms and enable a deeper understanding of the limitations of such algorithms.

The edge-detection method was found to be superior to the hHMM one for activities where there were many different interpretations such as skipping, or where the activity had more than one part such as stairs and stepping where up and down have distinctly different motion signals. However, this could be improved by adding more examples of the problematic activities or more fine-grained models for them.

Activities where the motions were more distinctive and the pressure edges were sharp, such as jumping, both methods perform similarly, although there is an increase in the F1-score. The hHMM method outperformed the edge-detection one for walking, jogging, and running activities, especially where longer steady-state sections existed, with respect to effort while with respect to the F1-score, the hHMM outperformed in all activities except stairs, hopping, skipping, and side-step.

The edge-detection and the cycle segmentation methods all additionally needed an energy threshold to distinguish between motion and rest. The generalizability of these parameters is limited as seen by the wide variance of required effort, see Batch 1 in Figure 6a. Furthermore, the edge-detection method requires clear edges within the pressure data and minimal data loss, which was not the case for the data collected in Germany. The hHMM-based method has the capacity to detect several different activities and is useful for detecting rest and activity without the need for manual selection of parameters. However, it cannot be used in isolation and needs some training data to start the initial training iteration. The high effort of the hHMM seen for activities such as hopping and stairs could be reduced with more data and optimized features, which would also potentially allow an unrestricted prediction reducing the effort further. Alternatively, the effort could be further lowered by including personalized models.

For cycle only detection, the PD and LCE methods were equally useful. However, for less clear activities than cycling, LCE should be superior due to its reliance of cyclicity and not only on peaks, although it would still need a method for rest and activity classification.

The combination of these methods reduced the overall effort of labeling the complete dataset to 16.3%, with a miss rate of 10.4% and a false detection rate of 10.5% and the labeling of new data using the current model can be expected to require only 14.1% effort overall and an F1-score of 93.0%. For datasets dominated by different ranges and locations of walking or jogging bouts the labeling effort would be under 10% with an overall F1-score of over 95%. In future applications a semi-supervised or real time approach for task labeling could be implemented. Currently the pipeline used labelers familiar with IMU-based motion data; however, the authors believe that because over 80% of the labels suggested were correct, a labeler unfamiliar with such data would have a sharp learning curve.

## 6. Conclusions and Outlook

Medical applications of cyclic activity monitoring such as step-counting and gait analysis are currently limited by a lack of realistic and labeled datasets. We provided a smart annotation pipeline which reduces the percentage of labels which need manual adjustment to 14%, which has enabled the production of a public dataset with over 150,000 labeled cycles, each with 2 phases from 80 participants. The dataset is diverse with 12 activities, 10 of which are cyclic; it includes transitions, ranges of bouts, and non-straight walking. For datasets such as home monitoring, where mostly walking data is expected, the labeling effort of new datasets can be expected to be as low as 8%. We believe that the smart annotation as well as the dataset will be instrumental in benchmarking other semi- and unsupervised learning algorithms as well as simultaneous segmentation and classification algorithms.

In this paper, we have proposed the use of an iterative training technique for a hHMM. The hierarchy of this model now includes cycle phases for each of the 10 cyclic activities. This method enabled the dataset made public in [16] to be increased 4 times with a final miss rate of 0.6% and false discovery rate of 7.6%. The complete pipeline achieves an F1-score of 89.5% with an expected accuracy for new data of F1 score 93.0%. With this pipeline existing datasets will be able to be increased with far less labeling effort and the current dataset and pipeline can be used as a benchmarking tool for further smart annotation pipelines focusing on cycle analysis in human motion.

## Figures and Tables

**Table 1:** Participant characteristics of publicly Available Wearable-based Cyclic Activity Recognition Databases (Healthy subjects).

Dataset Name	No. of Subjects	Duration	Cyclic Labels
MAREA [9]	20	34 min per subject	Heel strike, toe off (using FSR)
Bradjic [29]	27	Unknown	Step count (from video)
CMU-MMAC [5]	43	5 recipes	N/A
MHAD [7]	12	82 min (total)	N/A
OU-ISIR2012 [10]	744	Under 20 steps per subject	N/A
ZJU-GaitAcc [30]	175	6 times 20 m	Cycle borders (Signal analysis)
Real World (HAR) [28]	15	70 min per subject	N/A
Digital Biobank [33]	70	40 m plus 2 times 2 min	Cycle borders (Signal analysis)
Kluge [8]	15	1166 strides	Heel strike, toe off, heel off
			(Mocap)
Dailiac [6]	23	20 min	N/A
Martindale [31]	18	3500 strides	Cycle borders
BASA [32]	15	20 min per subject	Step count
FAU-Gait (current paper)	80	20 min per subject	Heel strike, toe off
(extending [16])			(smart annotation)

**Table 2:** Sensor and activity details of publicly Available Wearable-based Cyclic Activity Recognition Databases (Healthy subjects).

Dataset Name	Sensors and Sensor Location	*Activity Labels*
MAREA [9]	FSR, Acc (128 Hz)	Treadmill & outdoors,
	Left wrist, ankles, waist	Walk & run, slope & flat
Bradjic [29]	Acc, Gyr, Mag (100 Hz)	Slow, normal & fast
	Phone in pockets/hand/bag	straight walk
CMU-MMAC [5]	Acc, Gyr, (60 Hz) Mocap, Audio	Cooking
	Back, legs & arms	
MHAD [7]	Acc (30 Hz), mocap	11 actions: Jump, clap
	Wrists, ankles, hips	Throw, wave, punch, ...
OU-ISIR2012 [10]	Acc, Gyr, (100 Hz)	Level walking
	Waist	Slope & stair
ZJU-GaitAcc [30]	Acc (100 Hz)	Walk
	Arm, wrist, waist, ankle, thigh	
Real World (HAR) [28]	Acc, Gyr, GPS, Mag, Light, Sound	Walk, run, sit, stand,
	Chest, forearm, head, shin,	Lie, stairs, jump
	Thigh, upper arm, and waist	
Digital Biobank [33]	Acc, Gyr (100 Hz)	Straight walk
	Shoes	
Kluge [8]	Acc, Gyr (100 Hz)	Level walk
	Shoes	
Dailiac [6]	Acc, Gyr (100 Hz)	13 activities: Sit, lie,
	Shoes, chest, hip, wrist	Walk, stair, treadmill, skip ...
		Indoor & outdoors
Martindale [31]	Acc, Gyr (200 Hz)	Walk, run, stand
	Ankles	Outdoors
BASA [32]	Acc, Gyr (200 Hz)	Stairs, walk jog,
	Shoe, wrist	Sit, lie, stand
FAU-Gait (current paper)	Acc, Gyr (200 Hz), Pressure insole	Walk, jog, run, stand,
(extending [16])	Shoes, wrists, pocket	Jump, hop, skip, cycle, ...

Abbreviations: Accelerometer (Acc), Gyroscope (Gyr), Magnetometer (Mag), Force sensitive resistor (FSR), Motion capture (mocap).

**Table 3:** Subject characteristics.

Characteristic	Unit	Mean and Variance
Age	[years]	27 ± 6
Gender	[F/M]	28/52
Height	[cm]	174 ± 7
Weight	[kg]	66 ± 18
Shoe size	(EU)	41 ± 2
Handedness	[R/L]	74/4
Location	[Erlangen/Ljubljana]	56/24

**Table 4:** Tasks with corresponding activity labels; indicating if activity is cyclic and if it includes phases.

Task	Cyclic Activities with Phase Labels	Non-Cyclic Activities
Walk 2 × 20 m	Walk	Rest
Walk slalom	Walk	Rest
Walk circuit	Walk	Rest
Walk between activities	Walk	Rest
Sign name on posters	Walk	Rest
Jog 2 × 20 m	Jog	Rest
Jog circuit	Jog	Rest
Run 2 × 20 m	Run	Rest
Run slalom	Run	Rest
Run circuit	Run	Rest
Sitting at tables	Walk	Sit, Rest
Stepping	Stairs	Rest
Stairs	Stairs	Rest
Double jump	Jump	Rest
Jumping	Jump	Rest
Jumping on spot	Jump	Rest
Side-stepping	Side-step	Rest
Hopping	Hop	Rest
Skipping	Skip	Rest
Cycling	Cycle (no phase labels)	Rest
Running on spot	RunOnSpot	Rest

**Table 5:** Duration and quantity of activities.

Activity	Mean Duration ${}^{1}$	Mean Cycle Count ${}^{1}$	Total Cycles ${}^{2}$
Walking	523 s ± 63 s	492 ± 56	78,684
Jogging	60 s ± 12 s	78 ± 17	12,475
Running	55 s ± 14 s	85 ± 18	13,592
Stairs	65 s ± 11 s	67 ± 6	10,709
Jumping	40 s ± 6 s	77 ± 8	12,333
Hopping	11 s ± 2 s	12 ± 2	1932
Skipping	14 s ± 4 s	27 ± 10	4389
Side-step	9 s ± 2 s	16 ± 3	2653
Run on spot	16 s ± 2 s	21 ± 1	985
Cycling	62 s ± 10 s	85 ± 18	9482
Sit	120 s ± 34 s	-	-
Rest	397 s ± 106 s	-	-
Total	1343 s ± 137 s	983 ± 67	157,340

${}^{1}$ calculated per person, on the left foot data; ${}^{2}$ total for all subject.

**Table 6:** Mean of the labeling effort per activity, over all subjects in a given batch. Effort being the percentage of labels added or removed during manual labeling.

Activity	Overall Effort [%]	Effort Using EdgeDet [%]	Effort Using hHMM [%]
	All 7 Batches	Batch 1	Batch 7
All	16.3 ± 4.3	16.0 ± 5.3	14.1 ± 1.8
Walking (total)	12.5 ± 5.7	16.8 ± 6.1	8.5 ± 1.8
- circuit	6.2 ± 8.9	12.6 ± 11.4	0.8 ± 0.6
- 2 times 20 m	7.1 ± 8.9	14.6 ± 11.4	2.2 ± 1.4
- slalom	7.9 ± 10.4	15.9 ± 15.4	2.2 ± 2.0
- between activities	11.0 ± 6.2	16.4 ± 6.0	7.6 ± 3.1
- posters	38.7 ± 17.6	51.5 ± 14.1	23.5 ± 9.1
- between sitting	81.2 ± 40.7	45.4 ± 12.6	83.5 ± 47.9
Jogging (total)	9.1 ± 8.4	14.5 ± 9.4	4.9 ± 2.1
- circuit	7.6 ± 8.2	13.0 ± 9.3	1.2 ± 0.6
- 2 times 20 m	12.9 ± 11.7	18.1 ± 13.5	15.7 ± 5.3
Running (total)	15.5 ± 8.1	18.0 ± 9.4	14.9 ± 11.3
- circuit	7.9 ± 9.7	17.5 ± 12.4	1.2 ± 0.7
- 2 times 20 m	22.7 ± 14.4	26.2 ± 17.0	20.4 ± 12.8
- cones	25.4 ± 19.2	10.6 ± 7.7	43.1 ± 28.8
Stairs (total)	38.7 ± 18.9	18.4 ± 10.0	49.4 ± 14.9
- stepping	35.3 ± 22.7	17.0 ± 13.8	36.3 ± 18.3
- stairs	44.0 ± 24.2	20.3 ± 10.5	70.8 ± 23.3
Jumping (total)	12.4 ± 10.7	9.5 ± 7.6	8.1 ± 2.5
- jumping jacks	7.7 ± 14.2	6.6 ± 6.8	2.6 ± 1.9
- jumping on spot	14.7 ± 23.8	13.3 ± 17.6	3.7 ± 2.9
- double jump	20.7 ± 15.1	12.3 ± 9.5	24.1 ± 10.4
Hopping	46.3 ± 29.6	27.0 ± 21.5	56.7 ± 28.1
Skipping	26.2 ± 32.6	9.9 ± 9.5	23.8 ± 15.2
Side-step	47.1 ± 37.5	12.6 ± 8.8	40.9 ± 14.3
Run on spot	12.3 ± 12.4	12.3 ± 12.4	-
Cycle ${}^{1}$	2.9 ± 3.7	-	-

${}^{1}$ Using the peak-detection method.

**Table 7:** Mean of the F1 score per activity, over all subjects in a given batch.

Activity	Overall F1 [%]	F1 Using EdgeDet [%]	F1 Using hHMM [%]
	All 7 Batches	Batch 1	Batch 7
All	89.5 ± 4.8	83.8 ± 4.7	93.0 ± 0.8
Walking (total)	90.5 ± 7.5	80.5 ± 5.7	95.8 ± 0.9
- circuit	94.1 ± 8.7	84.4 ± 9.9	99.6 ± 0.3
- 2 times 20 m	96.4 ± 4.7	92.5 ± 6.1	98.9 ± 0.7
- slalom	96.0 ± 5.3	91.9 ± 7.8	98.9 ± 1.0
- between activities	90.0 ± 9.5	76.7 ± 6.0	96.2 ± 1.5
- posters	79.3 ± 11.1	68.2 ± 10.9	88.0 ± 4.6
- between sitting	65.3 ± 10.7	71.9 ± 9.1	67.2 ± 13.2
Jogging (total)	93.7 ± 6.8	86.9 ± 7.8	97.5 ± 1.1
- circuit	93.2 ± 8.7	83.4 ± 9.7	99.4 ± 0.3
- 2 times 20 m	93.5 ± 6.1	90.6 ± 7.2	92.1 ± 2.7
Running (total)	90.2 ± 6.2	84.3 ± 6.8	92.4 ± 5.8
- circuit	92.7 ± 9.8	80.2 ± 9.4	99.4 ± 0.4
- 2 times 20 m	88.3 ± 7.7	86.4 ± 9.0	89.5 ± 6.8
- cones	86.0 ± 10.1	91.5 ± 5.3	78.4 ± 14.6
Stairs (total)	79.0 ± 7.7	84.3 ± 6.6	75.6 ± 7.6
- stepping	81.5 ± 10.6	88.1 ± 8.1	81.9 ± 9.2
- stairs	75.2 ± 10.1	78.0 ± 8.2	65.4 ± 11.9
Jumping (total)	92.9 ± 5.9	92.5 ± 5.2	95.9 ± 1.2
- jumping jacks	95.1 ± 9.6	94.8 ± 4.7	98.7 ± 0.9
- jumping on spot	92.4 ± 11.1	91.5 ± 9.4	98.2 ± 1.4
- double jump	89.0 ± 7.0	90.6 ± 6.2	87.9 ± 5.2
Hopping	77.4 ± 11.8	82.1 ± 9.6	72.9 ± 13.3
Skipping	87.2 ± 13.4	92.5 ± 6.2	88.2 ± 7.5
Side-step	76.8 ± 14.7	90.3 ± 5.2	79.7 ± 7.5
Run on spot	90.9 ± 7.4	90.9 ± 7.4	-

**Table 8:** Mean of the miss rate (MR) and false discovery rates (FDR) per activity, over all subjects in a given batch.

Activity	Overall	Overall	MR [%]	FDR [%]	MR [%]	FDR [%]
Activity	MR [%]	FDR [%]	EdgeDet	EdgeDet	hHMM	hHMM
	All 7 batches	All 7 batches	Batch 1	Batch 1	Batch 7	Batch 7
All	10.4 ± 6.5	10.5 ± 3.6	19.1 ± 5.1	13.1 ± 4.6	0.6 ± 6.4	7.6 ± 1.2
Walking (total)	9.3 ± 9.5	9.6 ± 5.7	22.6 ± 6.1	16.1 ± 5.8	0.8 ± 3.3	5.1 ± 1.4
- circuit	6.4 ± 9.1	5.4 ± 8.3	17.1 ± 9.9	14.1 ± 10.0	0.5 ± 0.5	0.3 ± 0.2
- 2 times 20 m	3.9 ± 5.7	3.3 ± 3.9	9.0 ± 7.7	5.9 ± 4.7	0.5 ± 1.2	1.0 ± 1.2
- slalom	4.1 ± 5.7	3.9 ± 5.0	9.1 ± 8.2	7.0 ± 7.5	1.0 ± 1.1	1.1 ± 2.2
- between activities	9.6 ± 11.1	10.2 ± 8.2	25.4 ± 6.6	20.9 ± 6.3	1.4 ± 3.2	4.3 ± 1.7
- posters	22.5 ± 16.1	16.2 ± 9.2	43.1 ± 12.0	12.9 ± 8.7	5.3 ± 13.5	10.2 ± 5.7
- between sitting	28.9 ± 11.9	34.1 ± 21.6	40.8 ± 10.3	6.9 ± 8.2	8.6 ± 20.7	40.0 ± 17.6
Jogging (total)	6.9 ± 7.6	5.7 ± 6.0	14.9 ± 8.8	11.0 ± 7.4	1.0 ± 3.0	2.0 ± 1.1
- circuit	7.5 ± 9.5	6.0 ± 8.2	18.7 ± 9.8	14.3 ± 10.0	0.4 ± 0.9	0.3 ± 0.3
- 2 times 20 m	6.9 ± 7.1	6.0 ± 5.4	11.0 ± 9.1	7.4 ± 6.1	2.6 ± 8.9	7.0 ± 2.9
Running (total)	11.1 ± 7.3	8.4 ± 5.3	18.3 ± 7.4	12.8 ± 6.7	6.4 ± 8.3	6.8 ± 5.2
- circuit	8.5 ± 11.1	5.9 ± 8.5	23.1 ± 10.0	16.0 ± 9.3	0.6 ± 1.1	0.1 ± 0.3
2 times 20 m	13.1 ± 9.1	10.1 ± 6.7	15.9 ± 10.7	10.9 ± 8.4	8.7 ± 12.2	8.6 ± 5.0
- cones	14.7 ± 11.8	13.1 ± 8.7	9.3 ± 6.4	7.5 ± 5.6	15.1 ± 21.6	21.7 ± 14.1
Stairs (total)	21.2 ± 7.6	20.3 ± 9.5	20.2 ± 8.5	10.2 ± 6.0	8.7 ± 23.3	25.5 ± 6.6
- stepping	19.0 ± 10.4	17.8 ± 11.5	14.9 ± 9.8	8.4 ± 7.2	9.7 ± 17.6	18.5 ± 8.8
- stairs	24.5 ± 11.3	24.2 ± 12.1	28.1 ± 10.7	13.7 ± 8.5	13.4 ± 32.5	36.5 ± 10.7
Jumping (total)	7.2 ± 7.4	6.7 ± 5.2	9.2 ± 6.1	5.6 ± 4.8	1.1 ± 3.9	4.2 ± 1.5
- jumping jacks	5.7 ± 11.9	3.4 ± 4.9	7.3 ± 6.7	2.9 ± 3.1	1.1 ± 1.2	1.4 ± 0.9
- jumping on spot	6.9 ± 11.0	7.9 ± 11.7	10.0 ± 10.7	6.5 ± 8.9	1.4 ± 1.0	2.6 ± 2.3
- double jump	9.7 ± 6.7	12.1 ± 8.3	9.8 ± 6.4	8.9 ± 7.0	5.1 ± 12.2	11.9 ± 5.5
Hopping	18.5 ± 12.2	25.2 ± 14.1	19.4 ± 12.9	15.1 ± 9.7	13.6 ± 24.1	29.5 ± 14.2
Skipping	11.0 ± 11.8	14.1 ± 15.2	7.6 ± 7.7	7.0 ± 6.8	7.0 ± 11.1	12.5 ± 8.0
Side-step	20.7 ± 14.4	25.1 ± 15.5	9.1 ± 6.9	10.0 ± 5.3	8.5 ± 19.3	21.0 ± 7.2
Run on spot	9.4 ± 7.5	8.6 ± 7.7	9.4 ± 7.5	8.6 ± 7.7	-	-

## Abbreviations

The following abbreviations are used in this manuscript:

hHMM	Hierarchical hidden Markov model
IMU	Inertial measurement unit
Acc	Accelerometer
AX	Accelerometer axial plane
Gyr	Gyroscope
GZ	Gyroscope sagittal plane
Mag	Magnetometer
FSR	Force sensitive resistor
Mocap	Motion capture
PD	Peak detection
LCE	local cyclicity estimation
